# A Pilot Study on the Evaluation of an Inpatient Glycaemic Management Protocol for Enteral Feeding in People with Diabetes

**DOI:** 10.3390/diseases14090312

**Published:** 2026-08-26

**Authors:** Shayna Xueli Lin, Di Zhang, Khee Ling Choo, Qinghua Tan, Puja Sharda, Nur Kalimallah Khairul Anwar, Xin Yi Hannah Luah, Zongwen Wee, Priscilla Chiam Pei Sze, Angela Koh Fang Yung, Sueziani Bte Zainudin, Ling-Jun Chen

**Affiliations:** 1 Sengkang General Hospital, 110 Sengkang East Way, Singapore 544886, Singapore; 2 Ascensus Health Specialist Centre, 101 Irrawaddy Road, Singapore 329565, Singapore

**Keywords:** inpatient glycaemic management, enteral nutrition, diabetes mellitus, hyperglycaemia, hypoglycaemia, glucose monitoring, diabetes education

## Abstract

Aims: International diabetes guidelines recommend inpatient glycaemic management protocols for bolus enteral feeding in people with diabetes to improve clinical outcomes. This study aims to evaluate before and after hospital-wide implementation of an inpatient bolus enteral feeding protocol: (1) the incidence of hyperglycaemia (>13.9 mmol/L) and hypoglycaemia (<4.0 mmol/L), (2) medication prescribing practices and capillary blood glucose monitoring and (3) health care professionals’ knowledge and confidence levels. Methods: We implemented an inpatient glycaemic management protocol for bolus enteral feeding developed by a multidisciplinary team of diabetes nurse educators and endocrinologists and approved by the institutional medical board in July 2024. This before-and-after quality improvement study was conducted over 3 months across eight inpatient wards. Adult inpatients were consecutively enrolled if they met the inclusion criteria: (1) a documented diagnosis of diabetes mellitus, (2) receiving bolus enteral feeding and (3) treatment with glucose-lowering medication. Patients listed as critically ill were excluded. Nurses working in the pilot wards were also recruited. Before implementing the protocol, diabetes nurse educators trained inpatient nurses on understanding and executing the protocol for administering capillary blood glucose monitoring and medications for patients with diabetes on enteral feeding. Nurses’ pre- and post-knowledge levels and perceived confidence were assessed using a structured questionnaire. Electronic medical records were reviewed to evaluate the incidence rates of hypoglycaemia and hyperglycaemia before and during the 3 months following protocol implementation. We also assessed adherence to protocol-recommended capillary blood glucose monitoring frequencies based on the diabetes medication regimen. Results: A total of 31 patients were observed during the 6-week baseline period and 28 patients following protocol implementation. A total of 192 clinical care episodes were audited, comprising 78 in the pre-intervention phase and 114 in the post-intervention phase. The incidence of hyperglycaemia decreased from 43.6% to 10.5%, while hypoglycaemia decreased from 3.8% to 2.6%. After adjusting for protocol adoption rates, protocol implementation was associated with significantly lower odds of hyperglycaemia (odds ratio [OR] 0.22, 95% CI [0.07, 0.65], *p* = 0.006). A significant increase in appropriate nursing practices was observed post-intervention (*p* < 0.001). Adoption of the protocol by nurses decreased the odds of hyperglycaemia by 70% (*p* = 0.014). Nurses’ knowledge scores improved significantly from baseline to 3 months post-implementation (*p* < 0.001). Conclusions: Implementation of a standardised inpatient glycaemic management protocol for PWD receiving bolus enteral feeding was associated with reduced rates of hyperglycaemia and hypoglycaemia. Larger-scale studies are warranted to evaluate the effectiveness and sustainability of wider implementation in improving clinical outcomes.

## 1. Introduction

Bolus enteral feedings are commonly prescribed for hospitalised patients to meet their nutritional requirements [1]. For people with diabetes (PWD), such a feeding regimen increases risks for acute diabetes complications such as hyperglycaemia and hypoglycaemia [2]. Studies estimated that over 30% of PWD on bolus enteral feeding experienced significant hyperglycaemia [3]. Hyperglycaemia is associated with worse clinical outcomes, including poor wound healing and increased infection risk [4]. Conversely, hypoglycaemia can result from a mismatch between enteral feeding schedules and insulin administration, adding complexity to inpatient glycaemic management [5,6].

Pre-disposing factors and causes of hyperglycaemia and hypoglycaemia in patients receiving bolus enteral feedings are complex [7,8]. Optimising inpatient glycaemic control is challenging and is further aggravated by misaligned inpatient processes, such as overcorrection of hyperglycaemia with insulin or oral glucose-lowering agents (OGLAs), which can lead to hypoglycaemia [9]. In addition, apparent hyperglycaemia may result from capillary blood glucose (CBG) monitoring at inappropriate time points (such as immediately post-feeds) and/or medications administered at inconsistent or inappropriate feed timings.

In Singapore, physicians prescribe medication dosing and CBG monitoring frequency as twice or thrice daily (BD or TDS), while dietitians schedule bolus enteral feeds on time-based intervals (such as four bolus feeds/day at 0800, 1200, 1600, 2000 h). The subjective interpretation of medical orders for schedules with multiple feeding times creates ambiguity and variability in execution among healthcare professionals. This is further compounded by compartmentalisation of roles in a functional nursing care model, with different nurses performing tasks such as CBG monitoring, administering medications, and serving bolus enteral feedings. Inadvertently, deciphering of these instructions varies, leading to inappropriate or erroneous time points of CBG monitoring and/or medication served inconsistently, resulting in hyperglycaemia or hypoglycaemia. This practice is similar in countries such as Taiwan and Korea, where nurses rely on their own experiences or those of co-workers when administering enteral medications, leading to inconsistency [10,11], while in Japan, continuous enteral feedings with a twice-daily insulin regimen are being practised [12].

To date, meta-analyses exploring different treatment regimens have found no significant difference between strategies, yet formula feeds with a lower glycaemic index have been proposed [2]. While there are no clear recommendations on timings of administration for feeds, the American Association of Clinical Endocrinologists (AACE) and American Diabetes Association (ADA) strongly advocate for protocols to optimise management of inpatient glycaemia via coordinated medication administration with bolus enteral feeds timings for hospitalised PWD with a system-wide strategy for consistency [13]. Despite endorsement by the Joint British Diabetes Societies for Inpatient Care (JBDS-IP), a United Kingdom survey found that only 42% of hospitals had established guidelines [14,15].

Given this background and the concerning need for a standardised clinical approach, a novel protocol with defined steps has been developed to guide prescribers and nurses on the timing of CBG monitoring, administration of OGLAs and insulin for PWD on bolus enteral feedings. This formal protocol is unprecedented in Singapore and, unlike existing publications or guides, includes explicit steps and instructions for CBG monitoring and the types of OGLAs and insulin medications based on various bolus enteral feeding regimens.

## 2. The Study

### 2.1. Aims and Objectives

This study aims to evaluate the clinical outcomes of implementing an inpatient glycaemic management protocol for PWD on bolus enteral feeding as measured by acute diabetes complications and nurses’ knowledge level. The specific outcomes evaluated before and after the protocol implementation were

(1) The proportion of appropriate ordering of CBG monitoring by doctors and performance of CBG monitoring and administration of medication by nurses.
(2) The episodes of hyperglycaemia and/or hypoglycaemia within 24 h after insulin and/or OGLA administration, based on protocol guidelines.
(3) The level of nurses’ knowledge and confidence related to management of PWD on enteral bolus feedings.

### Definitions

For this study, we defined hyperglycaemia as >13.9 mmol/L and hypoglycaemia as <4.0 mmol/L, in accordance with Singapore Ministry of Health (MOH) clinical practice guidelines for diabetes [16], which are aligned with international standards [17]. Each occurrence of hypoglycaemia and hyperglycaemia within 24 h after administration of insulin and/or OGLAs was considered an incident episode.

Clinical care processes in this manuscript refer to the management of patients with diabetes receiving bolus enteral feedings, specifically physicians’ prescriptions of CBG timings and insulin and/or OGLAs and nurses’ performance of CBG monitoring and administration of insulin and/or OGLAs.

### 2.2. Methodology

#### 2.2.1. Study Setting and Sampling

This before-and-after study was conducted in Sengkang General Hospital (SKH), one of the acute tertiary 1000-bed hospitals in Singapore.

Eight inpatient wards, comprising four medical and four surgical wards, were studied 6 weeks before and 3 months after protocol implementation, from April 2024 to September 2024. Patients meeting the inclusion criteria—(1) adults with a known diagnosis of diabetes mellitus, (2) receiving enteral bolus feeding, and (3) receiving diabetes treatment—were enrolled. Patients who were on the “Dangerously Ill List” (DIL) were excluded. The study team recruited PWD on bolus enteral feedings upon patients’ admission to the pilot wards. During baseline data collection and the post-intervention phase, the clinical care processes carried out on these patients were audited. Nurses working in the piloted wards were also recruited.

In this hospital, inpatient nursing care comprises a hybrid of team nursing and functional nursing care models. Registered nurses (RNs), mostly without specialised training, oversee patient care, including medication administration, while enrolled nurses (ENs) or other nurse aides provide bedside nursing care such as CBG monitoring and enteral bolus feedings. A specialised diabetes department consisting of 5 diabetes nurse educators (DNEs), 1 endocrinology Advanced Practice Nurse (APN), and 11 endocrinology consultants support more complex diabetes inpatient management via referral or upon request.

#### 2.2.2. Study Intervention

**Figure 1:**
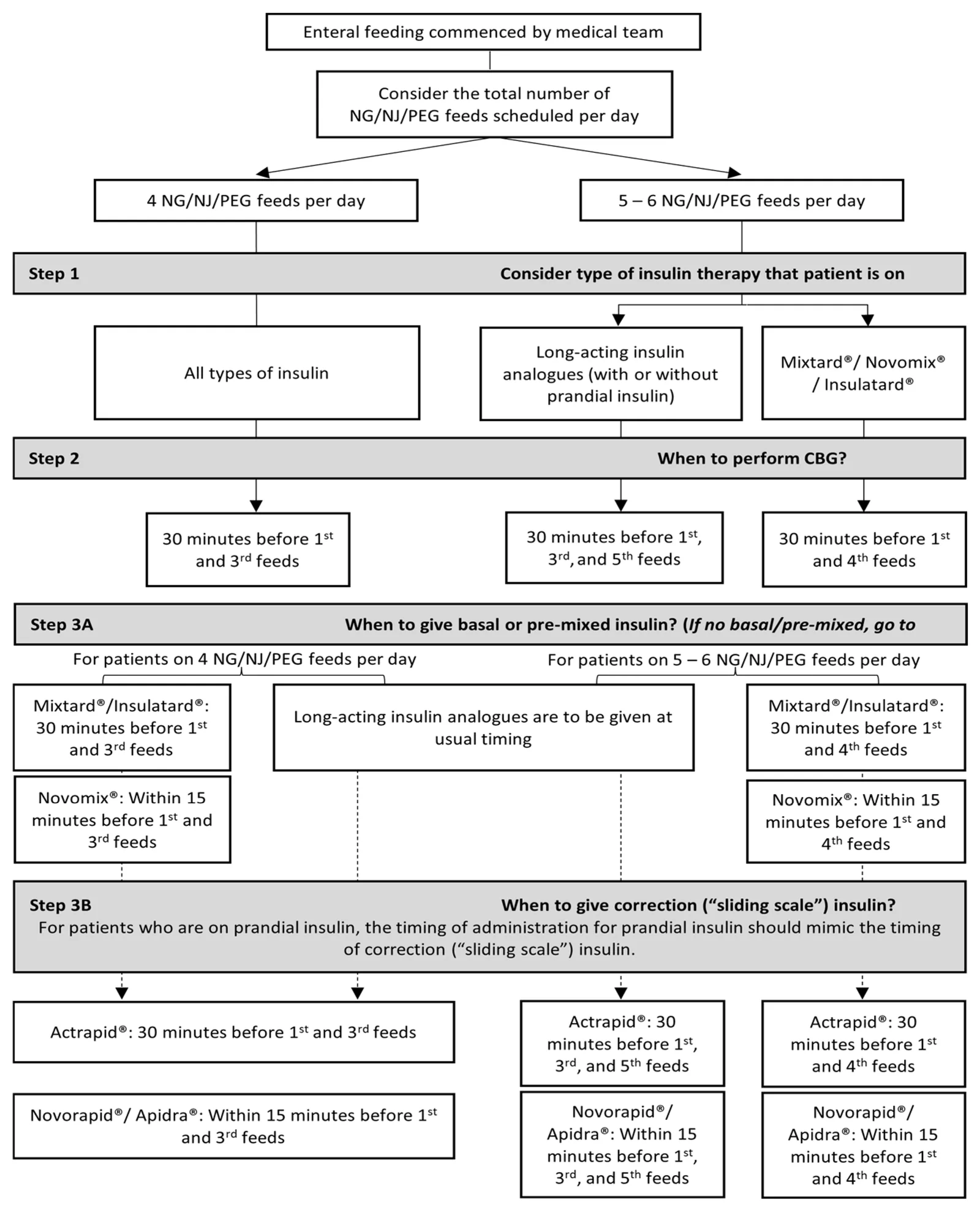
Protocol guideline on glycaemic management of patients with diabetes mellitus on bolus enteral feeding with insulin therapy.

**Figure 2:**
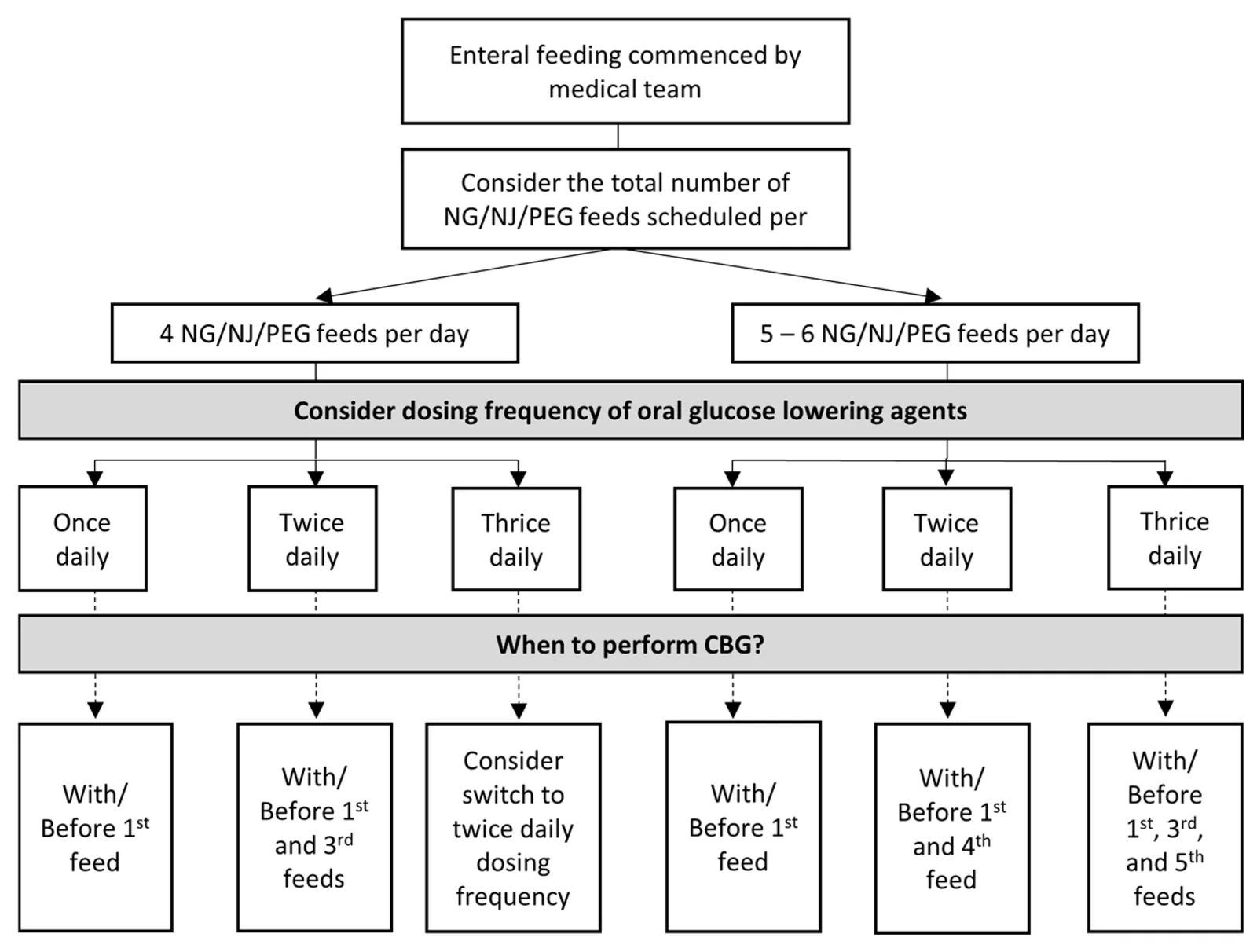
Protocol guideline on glycaemic management of patients with diabetes mellitus on bolus enteral feeding with oral glucose lowering agents.

The DNEs and endocrinologists developed a clinical protocol (Figure 1 and Figure 2) based on the core principles outlined in recommendations by AACE, ADA, and JBDS [18,19]. It aimed to standardise (1) the timing of CBG monitoring, (2) insulin and/or OGLA administration, and (3) modification of diabetes treatment and/or correctional insulin prescription based on the frequency of feeds (4, 5 or 6 feeds per day).

The protocol is a step-by-step guide designed to help physicians select the bolus feeding regimen ordered by dietitians and the initial OGLA or insulin therapy. It also delineates the precise timing required for CBG monitoring and subsequent therapeutic administration, including corrective insulin doses (Figure 1 and Figure 2). This standardises the prescribing instructions and guides nurses in the execution of CBG and medication administration.

**Figure 3:**
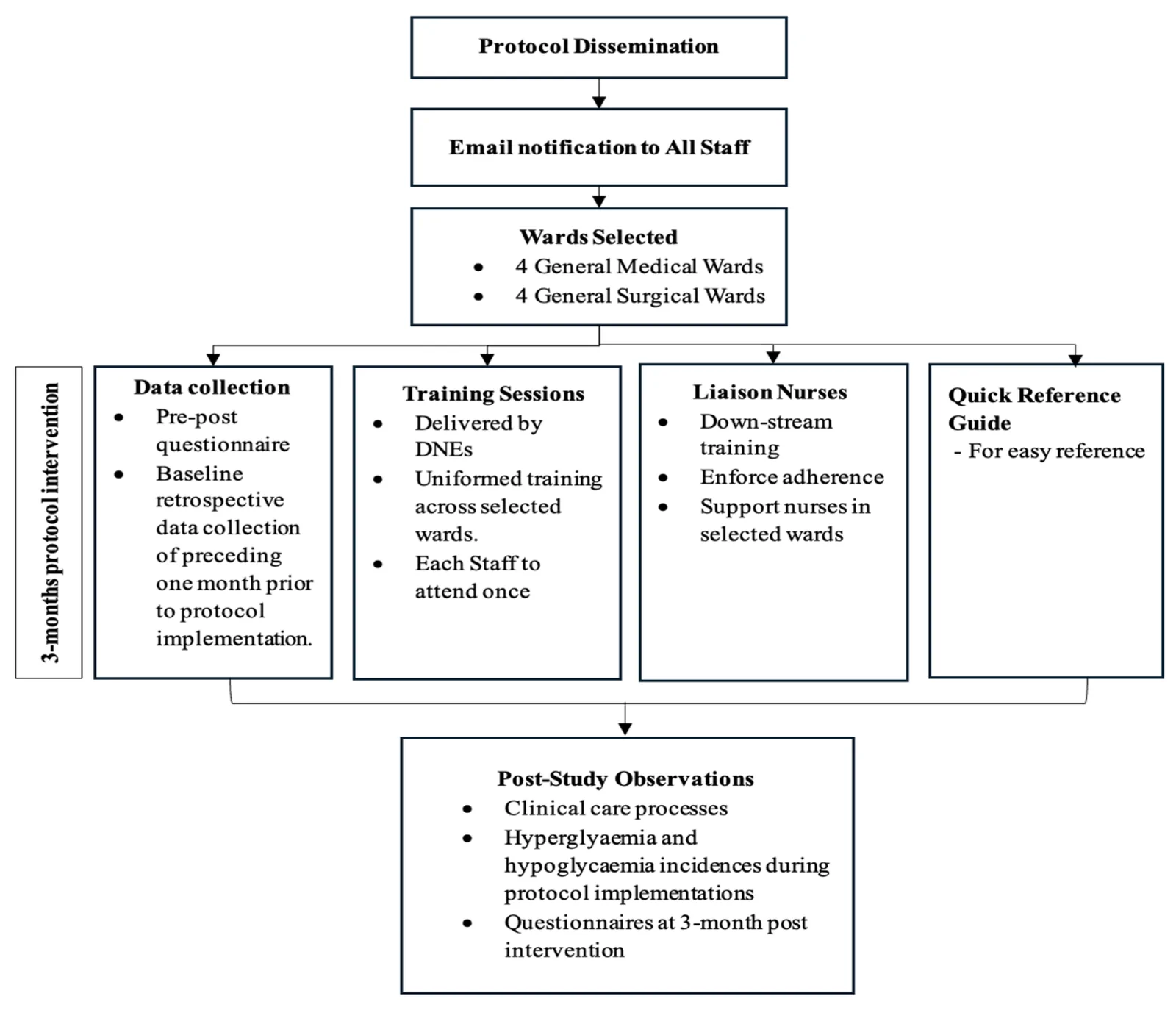
Study flow diagram.

The hospital protocol was disseminated via email to all hospital healthcare professionals, followed by a training session by DNEs for each selected ward. The training content included teaching of inpatient glycaemic thresholds for intervention for hypoglycaemia and hyperglycaemia and understanding and utilising the protocol. A quick reference simplified infographic guide was provided for immediate bedside reference. The study is represented in a flow diagram (Figure 3). All RNs and ENs were scheduled to attend the session at least once. Each ward appointed “liaison nurses” to remind and enforce adherence to protocol per shift.

### 2.3. Data Collection

#### 2.3.1. Instruments

Electronic medical records (EMRs) were reviewed for baseline patient demographics (age and gender), presence of a diabetes-related admission diagnosis, and feeding regimen (4, 5, or 6 times a day). As this study explored the protocol’s effect of prescribing instructions on inpatient glycaemia levels, data on patients’ baseline glycaemic control (e.g., HbA1c) and other pre-existing medications (such as corticosteroids) were not collected, as they were deemed not relevant in this context.

A structured questionnaire was also administered to evaluate the outcomes of the protocol implementation. The questionnaire was developed and content validated by an APN director, senior nurse educators, DNEs and endocrinologists. Demographic data, including designation (RN, EN), years of experience (<3, 3–10, >10 years), and department discipline (medical, surgical), were obtained anonymously. The questionnaire assessed knowledge level and self-reported confidence levels; the knowledge assessment section consisted of nine multiple-choice questions assessing factors that determine CBG monitoring, timing of administration of different types of insulin or OGLAs, and administration of correctional insulin scales in relation to the enteral feeding regimen. Nurses’ knowledge level was scored by computing the proportion of correct responses and expressing it as a percentage. The last item was a binary-choice question, involving a “yes” or “no” response to whether the participants felt confident in the glycaemic management of patients with diabetes receiving enteral feeding.

#### 2.3.2. Baseline Data Collection

To examine the baseline proportion of appropriate CBG ordering by physicians, CBG monitoring performance, and medication administration by nurses, a prospective review was first conducted of the clinical care process of PWD on bolus enteral feedings through systematic EMR charts over 6 weeks before protocol launch. Hypoglycaemia and hyperglycaemia results of <4 mmol/L and >13.9 mmol/L respectively, were captured for data analysis. Each enrolled patient could have had more than one episode of clinical care process observed during the admission period.

#### 2.3.3. Post-Intervention Data Collection

After the protocol was launched, identical comprehensive analyses were conducted on PWD receiving bolus feedings admitted to these piloted wards. Doctors’ orders for CBG monitoring, nurses’ charted CBG performance, and OGLA and/or insulin administration were reviewed against the protocol to identify deviations. CBG results of PWD on bolus enteral feedings in the piloted wards were reviewed again after protocol implementation. All episodes of hypoglycaemia (<4 mmol/L) or hyperglycaemia (>13.9 mmol/L) within 24 h following documented administration of CBG monitoring, OGLA and/or insulin therapy were captured via EMR charts audit. This was done remotely on a daily basis, and random ad hoc face-to-face observations were also conducted by a study team member during the 3-month intervention. Duplicate entries of observations were eliminated.

Lastly, RNs and ENs attending the training session were voluntarily invited to complete a nine-item questionnaire electronically before protocol dissemination and at the end of the three-month intervention (Appendix A). Informed consent was implied upon questionnaire completion.

We used the SQUIRE reporting guideline to draft this manuscript, and the SQUIRE reporting checklist for editing (Appendix B) [20,21].

### 2.4. Data Analysis

Data were analysed using IBM SPSS Statistics (Version 27). Descriptive data are presented as mean and standard deviation (SD) for continuous variables and frequencies and percentages for categorical variables.

Patient demographics and characteristics were compared using the chi-square test and independent *t*-test. The chi-square test was used to compare doctors’ and nurses’ practices and the presence of hyperglycaemic and hypoglycaemic complications. To examine the association between protocol implementation and hyperglycaemic complication outcomes, while accounting for repeated measures within patients over time, we used Generalised Estimating Equations (GEEs) models. A binomial distribution with a logit link function was applied, and an autoregressive working correlation matrix was selected to account for within-cluster dependencies. All hypothesis tests, 95% confidence intervals and standard errors were calculated using robust estimators. The primary predictor across all models was protocol implementation status, adjusting for adherence among doctors and nurses.

Demographics of nurses who completed the pre- and post-intervention questionnaire were compared for homogeneity using the chi-square test. Pre- and post-intervention knowledge scores were compared using an independent *t*-test. Significance was set at a *p*-value < 0.05.

A priori sample size calculation on the number of care process episodes to evaluate protocol adherence was not performed because the study was undertaken as a quality improvement initiative. A post hoc assessment demonstrated that the sample size achieved exceeded the minimum total sample size of 88 care episodes. It was calculated based on the primary outcome of interest, which is the association between protocol adherence and glycaemic complications, using a medium effect size of 0.3, a statistical power of 80%, and a two-sided significance level of 0.05.

## 3. Results

### 3.1. Participants’ Characteristics

A total of 192 clinical care process episodes were observed, 78 episodes from 31 patients before protocol implementation and 114 from 28 patients post-intervention. Mean age of patients was 75.9 ± 10.4 years, and the majority (*n* = 37, 62.7%) were male. Only four patients were admitted for acute diabetes-related complications. There was no significant difference in the baseline characteristics of the two groups (Table 1). Feeding regimens were mostly five and six feeds/day, scheduled at equal time intervals between 0700 h and 2200 h.

### 3.2. Comparison of Appropriate Practice of Clinical Processes, Hypoglycaemia and Hyperglycaemia Outcomes

Doctors’ CBG ordering practices did not differ from baseline, whereas appropriate nursing practice improved significantly (*p* < 0.001) (Table 2). There was no significant difference in the incidence of hypoglycaemic episodes before and after protocol implementation. However, protocol implementation was associated with a significant reduction in hyperglycaemia, decreasing from 34 of 78 episodes (43.6%) in the pre-intervention period to 12 of 114 episodes (10.5%) post-intervention (*p* < 0.001).

All 192 observed episodes in the pre- and post-intervention groups were included in the GEE analyses. The multivariate analyses examined whether protocol intervention and the adherence of doctors and nurses to protocol could predict the odds of hyperglycaemia in PWD receiving enteral feeding (Table 3). The odds of PWD developing hyperglycaemia were reduced by 78% with the protocol intervention (adjusted odds ratio [aOR] = 0.22, 95% CI [0.07, 0.65], *p* = 0.006). Similarly, protocol-adherent nurse practice reduced the odds of hyperglycaemia in PWD by 70% (aOR = 0.30, 95% CI [0.11, 0.78], *p* = 0.014).

### 3.3. Comparison of Nurses’ Knowledge and Confidence

A total of 313 nurses completed the pre-intervention questionnaire, while 207 responded post-intervention (Table 4). For both pre- and post-intervention, the majority were female and registered nurses with less than 3 years of nursing practice. Table 5 shows the nurses’ knowledge scores and self-reported confidence levels in the glycaemic management of PWD on enteral feeding. Despite a significantly improved knowledge score post-intervention (*p* < 0.001), self-reported confidence remained unchanged. Further analysis showed a significant association between years of practice and self-reported confidence (*p* = 0.013). Nurses with more years of practice were more likely to report confidence.

## 4. Discussion

The positive findings from this project demonstrate that implementing this clinical protocol is associated with good patient outcomes, particularly reduced hyperglycaemia in our hospital setting. Compared with global reported inpatient hyperglycaemia rates ranging from 22% to 46% [1,22], our post-intervention rate of 10.7% is encouraging. More substantially, we achieved better glycaemic control without the increased risks of hypoglycaemia. Overall incidence of hypoglycaemia fell to 2.7% from a baseline of 3.8% in the pilot wards. This figure is lower than the reported incidence of 10.1% [23], suggesting steadier glucose levels with reduced glucose excursion and variability. The protocol effectively mitigated glucose variability, a documented independent predictor of mortality [24,25], demonstrating its capacity to enhance patient safety outcomes.

Efforts to reduce glucose variability in critically ill patients on continuous enteral feeding were also described in a single-patient study in which continuous insulin infusion was titrated based on 3-hourly CBG monitoring [26]. Glucose level stability was achieved from the fourth day of insulin infusion. Our study results demonstrate that, for non-critically ill patients, glucose level stability can also be achieved through concerted, pre-defined steps for managing PWD on bolus enteral feedings without intensive CBG monitoring and continuous insulin infusion.

The favourable outcomes observed with this protocol can be attributed to standardised clinical instructions and communication, which facilitated coordinated capillary blood glucose monitoring, medication administration, and correctional insulin delivered at consistent feeding intervals throughout the hospitalisation period. This created a concise glucose profile, enabling accurate interpretation and adjustment of medication. Simultaneously, hypoglycaemia was reduced because erroneous or excessive correction of glucose levels was effectively averted. Secondly, oral medication and insulin were administered at almost equal intervals corresponding to the feeding schedule, allowing a therapeutic glycaemic-lowering effect on enteral bolus feeds and achieving better glucose targets [1,22]. Third, CBG tests and correctional insulin were not administered on isolated feeds not served with scheduled glucose-lowering treatment, preventing hypoglycaemia from overcorrection. However, we acknowledge that unmeasured confounding variables, including expectation and observation bias, may have contributed to the observed positive outcomes.

We also note that adherence to this protocol was suboptimal, with nurses faring better at 50%. Mirroring the literature, adherence to type 2 diabetes guidelines was around 40% to 60% [27]. Time constraints in clinical settings, protocol complexity and novelty, and therapeutic inertia could account for non-adherence to protocols [28]. Given that the protocol is newly developed, some healthcare professionals may need time to adapt and integrate it into their clinical practice. In contrast, others may need to observe demonstrable outcomes before fully endorsing its implementation. Nonetheless, nurses’ adoption of the protocol has reduced hyperglycaemia by 74%, suggesting a likelihood of positive outcomes. Nurses’ adherence to the protocol for CBG monitoring and matched medication administration is a key factor in achieving targeted glucose levels and improving overall patient health outcomes. Nurses’ role as key players in multidisciplinary teams and as primary implementers of healthcare interventions for PWD amplifies their autonomy and responsibility to rectify medication orders when instructions fall short of standard care [29].

Additional implementation barriers included insufficient training for newly boarded nursing staff and doctors, inaccessible protocols, and perceived clinical complexity, which likely compromised fidelity and adherence. This was exacerbated by attenuated support and a lack of dedicated liaison nurses during night shifts and weekends to reinforce protocol compliance. Furthermore, the intervention coincided with the rotation of junior physicians within the pilot units, resulting in poor awareness and a concurrent decline in physician adherence to protocols.

An integrative review on improving nurses’ adherence to diabetes guidelines highlighted the importance of multi-pronged strategies comprising active methods, such as interactive discussion, training, and education, and passive methods such as reminders and education materials [30]. A combination of in-person update sessions, teaching, and practical scenario sharing helps integrate knowledge into practice. This multifaceted learning, reinforced with reminders and reading materials, is most effective in fostering more sustainable adherence to guidelines and protocols [31].

Although training is expected to build knowledge and strengthen self-confidence, a surprising outcome in this project was that nurses’ reported self-confidence level did not change after the intervention. This may explain the decline in post-intervention responses. A large multi-site study in Singapore also echoed a weak correlation between nurses’ knowledge of sepsis and self-confidence [32]. One possible explanation is that, because professional confidence is defined as an inner sense of self-confidence and calmness in clinical circumstances, the dynamic nature of executing protocol steps may be intimidating during initial induction [33]. Clinical execution of the protocol is challenging, involving communication and coordination with various colleagues. Unfamiliarity and apprehension of failure are also major hindrances to confidence [34]. Professional confidence was also highest among working nurses with 10 years or more of experience, affirming that adequate working experience is needed to develop competency and confidence [16,35]. Our study also found that nurses with more than 3 years of experience reported higher perceived self-confidence. Extended experience in the clinical setting is thus instrumental in helping nurses develop self-efficacy in managing diverse and complex care. Exposure to a wider range of patient care and conditions enables the accumulation of clinical competence and the development of sound clinical judgement when dealing with new care processes or protocols [16,35].

Improving self-confidence would therefore enhance competence and, in turn, further improve confidence, creating a progressive, positive spiral of improvement. Hence, it is crucial to integrate practical scenarios in training to advance the quality of education [36]. These examples should include real-life communication, interaction, and coordination with various healthcare professionals through an interactive role-playing model that mimics actual circumstances. This will foster familiarity and help eliminate apprehension about independent care practices.

### 4.1. Further Implementation Strategies

**Figure 4:**
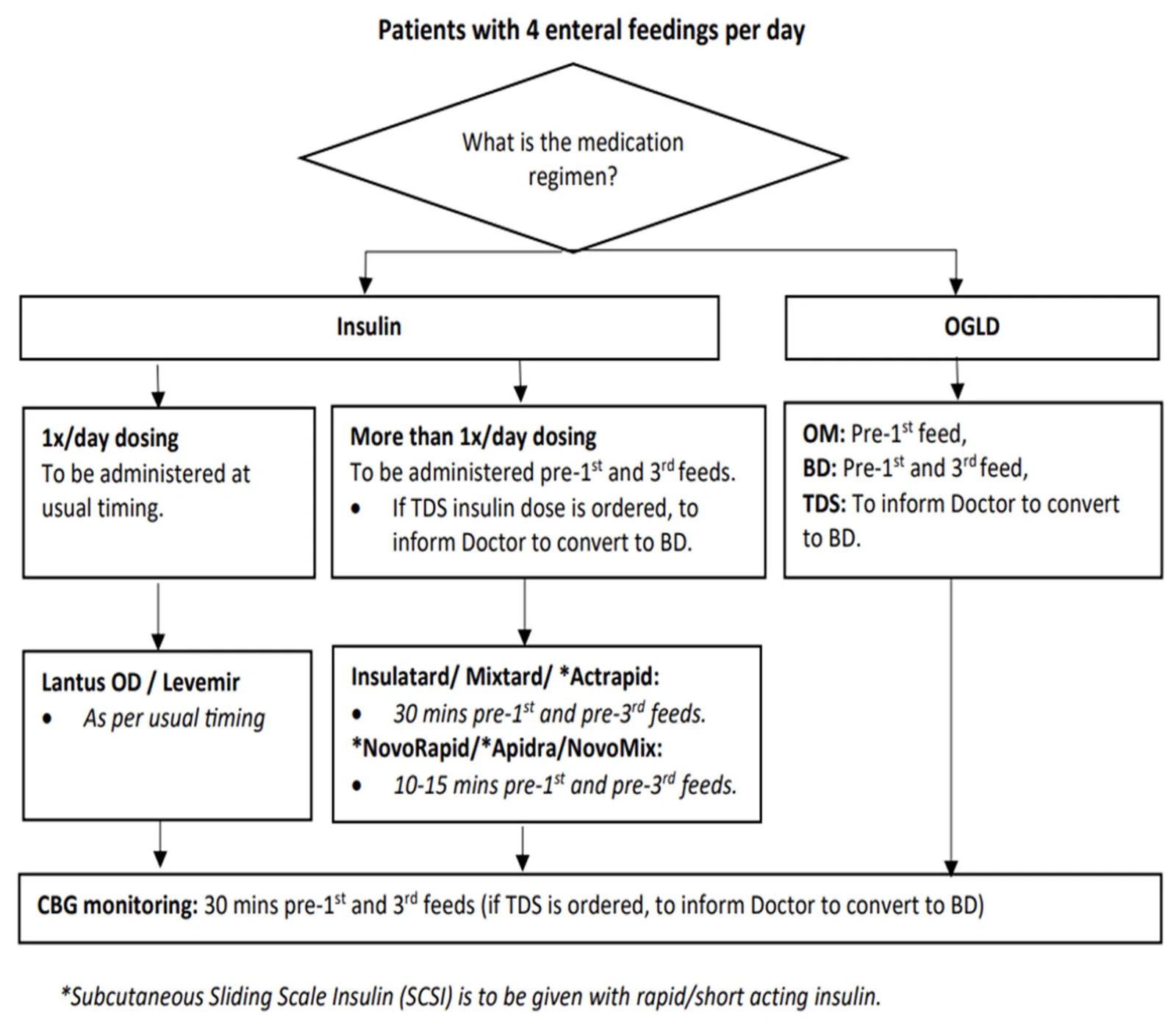
Nursing workflow—4 enteral feedings per day.

**Figure 5:**
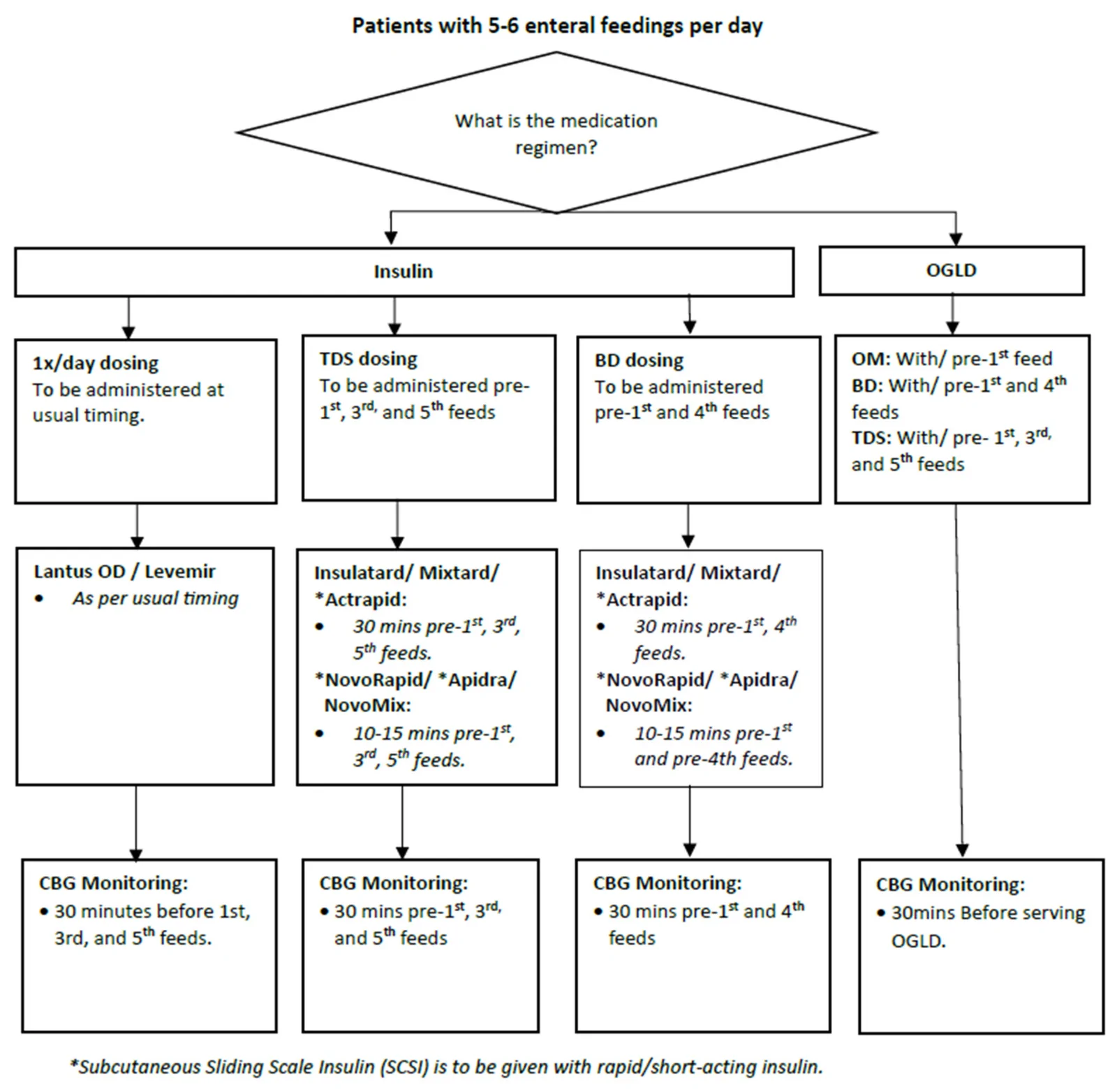
Nursing workflow—5–6 enteral feedings per day.

The success of the piloted wards showed that the protocol improved patient outcomes and clinical efficiency. This led to the decision to expand the protocol to other inpatient wards, allowing more patients to benefit from the positive results. The project team first developed strategies to improve implementation in other wards. Focus group discussions with nurses were conducted to deepen understanding of their experiences and to inform improvement strategies in the piloted wards. Nurses’ verbatim responses were mostly positive. Nurses deemed the protocol advantageous in standardising the care process and preventing confusion in practice. Prominent themes included poor awareness of the protocol among medical doctors, the need to refer to the protocol frequently, difficulty remembering the steps, and poor awareness among new hires. Based on nurses’ feedback, a simplified workflow was derived from the original protocol to improve adherence and understanding (Figure 4 and Figure 5). The quick reference guide underwent several revisions to optimise visual clarity, and training sessions were revamped with a new focus on understanding feeding and medication intervals. Revised versions were subsequently employed to train all inpatient ward lead nurses and clinical nurse instructors. This ensured proper training was disseminated to all.

The project’s analysis and results were shared with the heads of the departments of medicine, pharmacy, and dietetics. At the time of writing this manuscript, this clinical protocol has been integrated into doctors’ induction instruction manuals and e-learning training. Pharmacists and dietitians were also recruited as collateral partners to enforce adherence through physical documentation or communication with care team doctors or nurses.

### 4.2. Implications/Recommendations

The potential of this protocol in reducing glycaemic excursions and preventing adverse patient outcomes supports its use in managing patients with diabetes receiving bolus enteral feedings. Its impact on aligning instructions and practice among staff or carers will be most appropriate for long-term PWD relying on bolus enteral feedings. These include those in geriatric wards, community hospitals, and step-down care facilities such as nursing homes, where the guide could help standardise treatment regimens among healthcare professionals.

### 4.3. Limitations of Work/Recommendations for Future Research

This study has several limitations. First, its single-centre design limits its generalisability to a national level. Second, given the project design, causal inference about the protocol’s impact on reducing glycaemic complications cannot be made. The evaluation also did not account for physicians’ training or prior experience, which could have influenced protocol adherence and overall outcomes. Although the project team standardised remote observations and documentation audits, inter-rater reliability was not enforced for the clinical audit. The Hawthorne effect may have influenced the observed positive outcomes. Rigour in reducing response bias in knowledge acquisition was also not maintained, as answers could have been discussed while completing the questionnaires.

Future research would benefit from evaluating other key indicators, such as length of hospital stay and mortality rates, to reflect patients’ clinical outcomes better. Training should also include doctors and allied healthcare professionals, such as dietitians and pharmacists, who are involved in the direct care of patients receiving bolus enteral feeds. For sustainability, future studies should evaluate clinical outcomes over an extended period to more concretely assess the internal and external validity of the findings.

## 5. Conclusions

Managing hyperglycaemia is a challenge for healthcare professionals caring for inpatients with diabetes receiving multiple bolus enteral feedings. This quality improvement study demonstrated that implementing a standardised clinical protocol that aligns CBG monitoring and medication administration with enteral feeding schedules was an important factor in reducing hyperglycaemic complications. An integrated, multifaceted training session comprising physical teaching and passive reminders for all healthcare professionals is necessary to maximise protocol adoption. By enhancing healthcare professionals’ education on effective clinical protocols, patient care can be optimised to achieve quality health outcomes.

## Figures and Tables

**Table 1:** Characteristics of the patients whose care processes were observed before and after protocol implementation.

Demographics and Characteristics	Total (*n* = 59)	Before Implementation (*n* = 31)	After Implementation (*n* = 28)	*p*
Age, mean (SD), y	75.9 (10.4)	76.5 (11.3)	75.3 (9.5)	0.670
Male	37 (62.7)	21 (67.7)	16 (57.1)	0.401
Diabetes-related admission diagnosis	4 (6.8)	3 (9.7)	1 (3.6)	0.352
Feeding regime				0.614
4 times/day	13 (22.0)	6 (19.4)	7 (25.0)	
5 times/day	25 (42.4)	15 (48.4)	10 (35.7)	
6 times/day	21 (35.6)	10 (32.3)	11 (39.3)	

Values are number of older adults (%) unless indicated otherwise.

**Table 2:** Outcomes of protocol implementation on the practice of the healthcare providers and glycaemic complications.

	Before Implementation *n*/n (%)	After Implementation *n*/n (%)	*p*
Observed episodes demonstrating appropriate practice of the doctors and nurses			
Appropriate practice of the doctors	11/78 (14.1)	14/114 (12.3)	0.713
Appropriate practice of the nurses	20/78 (25.6)	57/114 (50.0)	<0.001
Observed episodes with hypo- or hyperglycaemia complication			
With hypoglycaemia	3/78 (3.8)	3/114 (2.6)	0.635
With hyperglycaemia	34/78 (43.6)	12/114 (10.5)	<0.001

**Table 3:** GEE model to evaluate variables predicting presence of hyperglycaemia complications.

					95% CI	
Variables	*b*	SE	Sig.	Exp(B)	Lower	Upper
Protocol implementation	−1.53	0.56	0.006	0.22	0.07	0.65
Appropriate practice of the doctors	−0.30	0.92	0.746	0.74	0.12	4.50
Appropriate practice of the nurses	−1.22	0.49	0.014	0.30	0.11	0.78

**Table 4:** Nursing staff demographics.

		Before Implementation (*n* = 313)	After Implementation—3 Months (*n* = 207)	*p*
		*n* (%)	*n* (%)	
Job designation	Registered nurses	230 (73.5)	162 (78.3)	0.216
	Enrolled nurses	83 (26.5)	45 (21.7)	
Years of practice	<3 years	164 (52.4)	112 (54.1)	0.847
	3–10 years	121 (38.7)	75 (36.2)	
	>10 years	28 (8.9)	20 (9.7)	
Practice discipline	Medical	141 (45.0)	97 (46.9)	0.685

**Table 5:** Knowledge scores and perceived confidence of the nurses.

		Before Implementation	After Implementation—3 Months	*p*
Knowledge score, Mean (SD)		5.22 (1.71)	5.87 (1.74)	<0.001
Perceived confidence, *n* (%)		126 (40.3)	80 (38.6)	0.714
Perceived confidence, *n* (%)	<3 years		33 (29.5)	0.013
	3–10 years		37 (49.3)	
	>10 years		10 (50)	

## Data Availability

The data presented in this study are available from the corresponding author upon reasonable request.

## Appendix A

1.	What is your profession?
2.	What is your role?
3.	Designation?
4.	How many years of experience?
5.	Which ward are you from?
6.	For patients on enteral feedings, CBG should be performed: a. Right before enteral feedings. b. 30 min before serving insulin regime, depending on insulin regime. (answer) c. Normal TDS timing, i.e., pre-breakfast, pre-lunch, pre-dinner. d. Check after feedings.
7.	Dr X has ordered “CBG TDS” for a patient on 4 enteral feeds per day. The patient has a history of diabetes. The patient is on glipizide 2.5 mg twice daily and is not on insulin therapy. You decide to clarify with Dr X on when exactly CBG should be performed. For a patient on 4 enteral feedings a day and not on insulin treatment, CBG should be performed at: Enteral feeding regimen: Fresubin 2 kCal Drink (Vanilla) Enteral 200 mL, (every 1 day 08:00, 12:00, 16:00, 20:00), 200 mL water flush a. Pre-1st, pre-2nd and pre-4th feeds, i.e., at 0800 h, 1200 h and 2000 h. b. Pre-1st, pre-3rd and pre-4th feeds, i.e., at 0800 h, 1600 h and 2000 h. c. At 0800 h, 1200 h and 1700 h as per usual TDS timings. d. Pre-1st and pre-3rd feeds, i.e., at 0800 h and 1600 h (answer).
8.	Dr Y has ordered CBG TDS for a patient on 5 enteral feeds per day. When should CBG be performed? Enteral feeding regimen: Diben Drink (Vanilla) Enteral 200 mL, (every 1 day: 08:00, 11:00, 14:00, 17:00, 20:00) 120 mL water flush post feed. a. At 0800 h, 1100 h and 1700 h as per usual TDS timings. b. Pre-1st, pre-3rd and pre-5th feeds. (answer) c. At any timing that is convenient. d. Before each enteral feeding, i.e., 5 times a day.
9.	Patient B is on the following NGT 4 feeding regime. Dr Y has specified the timings for SCSI Actrapid sliding scale as 0730 h, 1030 h, 1830 h. What would you do for this patient’s SCSI Actrapid and NGT feedings? Enteral feeding regimen: Glucerna Liquid Enteral 250 mL, (every 1 day: 08:00, 12:00, 16:00, 20:00) 30 mL water flush post feed. Sub-Cutaneous Insulin Sliding Scale Actrapid (Insulin Soluble) Injection Sub-Cutaneous 0 unit if Capillary Blood Glucose (mmol/L) 1−12 2 units if Capillary Blood Glucose (mmol/L) 12.1−14 4 units if Capillary Blood Glucose (mmol/L) 14.1−20 <User Schedule> (every 1 day: 0730, 1030, 1830) Inform Dr if HC < 4 or >20. a. I will administer insulin sliding scale based on the doctor’s orders. b. I will adjust the timing of enteral feeds to match the doctor’s orders. c. I will inform the team that the SCSI order does not match with patient’s enteral feeding regimen. (answer) d. I will omit the 1830 h insulin sliding scale.
10.	Patient C was previously on enteral feeding but is currently kept nil-by-mouth (NBM) because of a procedure. The team has ordered his SCSI Actrapid sliding scale (0800 h, 1700 h) as below. Select the appropriate answer: Sub-Cutaneous Insulin Sliding Scale Actrapid (Insulin Soluble) Injection Sub-Cutaneous 0 unit if Capillary Blood Glucose (mmol/L) 1−12 2 units if Capillary Blood Glucose (mmol/L) 12.1−14 4 units if Capillary Blood Glucose (mmol/L) 14.1−20 <User Schedule> (every 1 day: 0730, 1030, 1830) Inform Dr if HC < 4 or >20. a. The insulin sliding scale should be omitted as patient will not have enteral feeds. b. Administer the sliding scale as per ordered. c. As the patient is NBM, CBG monitoring is not required. d. I will inform Doctor to change CBG monitoring frequency and Actrapid sliding scale timing to Q6H. (answer)
11.	Dr Z has ordered sub-cutaneous Insulatard 18 units BD for a patient with 5 enteral feeds per day. When should sub-cutaneous Insulatard be administered? Enteral feeding regimen: Glucerna Liquid Enteral 200 mL, (every 1 day: 08:00, 11:00, 15:00, 18:00, 22:00) 50 mL water flush post feed a. Pre-1st and pre-3rd feeds. b. Pre-2nd and pre-4th feeds. c. Pre-1st and pre-5th feeds. d. Pre-1st and pre-4th feeds. (answer)
12.	Patient B is on regular Mixtard 20units BD and 4 enteral feeds per day. Which of the following is correct? Enteral feeding regimen: Glucerna Liquid Enteral 250 mL, (every 1 day: 08:00, 12:00, 16:00, 20:00) 100 mL water flush post feed a. Administer Mixtard at 0730 h and 1730 h. b. Administer Mixtard pre-1st and pre-3rd feeds. (answer) c. Administer Mixtard pre-2nd and pre-4th feeds. d. Administer Mixtard pre-1st and pre-4th feeds.
13.	Patient C is on Metformin 850 mg TDS and Glipizide 5 mg BD and has 5 feeds per day via NGT prescribed as below. Which of the following is correct? Enteral feeding regimen: Glucerna Liquid Enteral 200 mL, (every 1 day: 08:00, 11:00, 15:00, 18:00, 22:00) 50 mL water flush post feed a. Serve PO Metformin pre-1st feed, pre-3rd, pre-5th feed and PO Glipizide pre-2nd and pre-4th feed. b. Serve PO Metformin pre-1st feed, pre-3rd, pre-5th feed and PO Glipizide pre-1st and pre-5th feeding. c. Serve PO Metformin pre-1st feed, pre-3rd, pre-5th feed and PO Glipizide pre-2nd and pre-5th feeding. d. Serve PO Metformin pre-1st feed, pre-3rd, pre-5th feed and PO Glipizide pre-1st and pre-4th feeding. (answer)
14.	Patient D is on 6 enteral feeds a day. He is on SC Insulatard 12 units BD and SC Actrapid 4 units TDS. Insulin sliding scale is ordered for pre-1st and 4th feeding. Prior to the first feed of the day, patient D’s CBG was found to be 3.0 mmol/L. What is your next action? a. Continue regular enteral feeds and administer all insulin as ordered. b. Administer 1 sachet of dextrose via NGT. Re-check CBG 15 min later. (answer) c. Administer 1 sachet of dextrose via NGT and proceed with regular enteral feeds and insulin. d. Withhold all insulin in view of hypoglycaemia and continue enteral feeds.
15.	How often do you manage patients with diabetes who are on bolus enteral feeding? a. Almost never b. Once in a while c. Sometimes d. Often e. Almost always
16.	How often do you manage patients with diabetes who are on bolus enteral feeding AND ALSO on insulin therapy? a. Almost never b. Once in a while c. Sometimes d. Often e. Almost always
17.	How confident are you in managing patients with diabetes who are on bolus enteral feeding? a. Not at all confident b. Slightly confident c. Moderately confident d. Quite confident e. Extremely confident
18.	How confident are you in managing patients with diabetes who are on bolus enteral feeding AND ALSO on insulin therapy? a. Not at all confident b. Slightly confident c. Moderately confident d. Quite confident e. Extremely confident
19.	How confident are you regarding the availability of guidelines on managing diabetes patients who are on bolus enteral feeding? a. Not at all confident b. Slightly confident c. Moderately confident d. Quite confident e. Extremely confident

## Appendix B. The Scheme Reporting Checklist

	**Item Description**	**Location (or Reason for Not Reporting)**
Title and Abstract		
1. Title	Indicate that the manuscript concerns an initiative to improve healthcare (broadly defined to include the quality, safety, effectiveness, patient-centredness, timeliness, cost, efficiency and equity of healthcare).	Page 1
2. Abstract	A. Provide adequate information to aid in searching and indexing.	Page 1
	B. Summarise all key information from various sections of the text using the abstract format of the intended publication or a structured summary such as: background, local problem, methods, interventions, results, conclusions.	Page 1
Introduction		
3 & 4. Problem description & Available Knowledge	3. Nature and significance of the local problem. 4. Summary of what is currently known about the problem, including relevant previous studies.	Page 4
5. Rationale	Informal or formal frameworks, models, concepts, and/or theories used to explain the problem, any reasons or assumptions that were used to develop the intervention(s), and reasons why the intervention(s) was expected to work.	Pages 4–5
6. Specific aims	Purpose of the project and of this report.	Page 6
Methods		
7. Context	Contextual elements considered important at the outset of introducing the intervention(s).	Pages 6–7
8. Intervention(s)	A. Description of the intervention(s) in sufficient detail that others could reproduce it.	Pages 7–8
	B. Specifics of the team involved in the work.	Pages 7 and 8
9. Study of the Intervention(s)	A. Approach chosen for assessing the impact of the intervention(s).	Pages 7 and 8
	B. Approach used to establish whether the observed outcomes were due to the intervention(s).	Page 8
10. Measures	A. Measures chosen for studying processes and outcomes of the intervention(s), including rationale for choosing them, their operational definitions, and their validity and reliability.	Page 8
	B. Description of the approach to the ongoing assessment of contextual elements that contributed to the findings.	Page 8
11. Analysis	A. Qualitative and quantitative methods used to draw inferences from the data.	Page 9
	B. Methods for understanding variation within the data, including the effects of time as a variable.	Page 9
12. Ethical considerations	Ethical aspects of implementing and studying the intervention(s) and how they were addressed, including, but not limited to, formal ethics review and potential conflict(s) of interest.	Page 10
Results		
13 a & b. Evolution of the intervention and details of process measures	A. Initial steps of the intervention(s) and their evolution over time (e.g., time-line diagram, flow chart, or table), including modifications made to the intervention during the project. B. Details of the process measures and outcome.	Page 10–11
13 c, d & e Contextual elements and unexpected consequences	A. Contextual elements that interacted with the interventions.	Page 10–11
	B. Observed associations between outcomes, interventions and relevant contextual factors.	Page 11
	C. Unintended consequences such as benefits, harms, unexpected results, problems or failures associated with the intervention(s).	Not applicable. No unintended consequences.
13 e. Missing data	Details about missing data.	Page 11
Discussion		
14. Summary	A. Key findings, including relevance to the rationale and specific aims.	Page 11
	B. Particular strengths of the project.	Pages 11–12
15. Interpretation	A. Nature of the association between the intervention(s) and the outcomes.	Page 12
	B. Comparison of results with findings from other publications.	Pages 12–13
	C. Impact of the project on people and systems.	Page 12
	D. Reasons for any differences between observed and anticipated outcomes, including the influence of context.	Pages 11–13
	E. Costs and strategic trade-offs, including opportunity costs.	Not applicable. Not examined and not relevant to study.
16. Limitations	A. Limits to the generalisability of the work.	Page 15
	B. Factors that might have limited internal validity such as confounding, bias, or imprecision in the design, methods, measurement, or analysis.	Page 15
	C. Efforts made to minimise and adjust for limitations.	Page 15
17. Conclusion	A. Usefulness of the work.	Pages 14–15
	B. Sustainability.	Page 14
	C. Potential for spread to other contexts.	Pages 14–15
	D. Implications for practice and for further study in the field.	Pages 14–15
	E. Suggested next steps.	Pages 14–15
Other information		
18. Funding	Sources of funding that supported this work. Role, if any, of the funding organisation in the design, implementation, interpretation and reporting.	Page 15
